# Probiotics for Anxiety and Depressive Symptoms in Cancer: A Systematic Review of Animal and Human Studies with Mechanistic Insights

**DOI:** 10.3390/microorganisms14010051

**Published:** 2025-12-25

**Authors:** Zahra Amirkhanzadeh Barandouzi, Deborah Watkins Bruner, Yufen Lin, Hannah Choi, Layla R. Zeki, Tobi Akangbe, Amruta Epari, Hongjin Li

**Affiliations:** 1 School of Nursing, Emory University, Atlanta, GA 30322, USA; zahra.barandouzi@emory.edu (Z.A.B.);; 2 College of Nursing, University of Illinois Chicago, Chicago, IL 60612, USA

**Keywords:** anxiety, depression, depressive symptoms, cancer, probiotics, kefir

## Abstract

Probiotics have been increasingly evaluated for their potential effect on anxiety and depression through the modulation of the gut–brain axis. Individuals with cancer experience a high prevalence of these symptoms. However, the effects of probiotics and their underlying mechanisms in this population have not been systematically evaluated. This review synthesizes current evidence regarding probiotic interventions for anxiety and depression in cancer and examines the associated mechanistic pathways. A systematic search for original trials in PubMed, Embase, CINAHL, Web of Science, and PsycINFO was conducted in May 2025. Eligible studies included animal models or adults with cancer who received probiotics alone or in combination with other treatments, with outcomes related to anxiety, depressive symptoms, or depression. Search terms included animal model, cancer, probiotics, anxiety, depressive symptoms, depression, gastrointestinal microbiome, gut microbiome, and microbiota. The review followed PRISMA guidelines. Risk of bias in trials was assessed using the SYRCLE and Cochrane RoB2 tool. Nine studies met the inclusion criteria, including seven human studies, one animal study, and one mixed human–animal study, with human sample sizes ranging from 24 to 266. The animal study reported reductions in depressive and anxiety-like behaviors, paralleled by modulation of the hypothalamic–pituitary–adrenal (HPA) axis, reduced inflammation, rebalancing of the gut microbiota, and improvements in neurotransmitter pathways. Findings from human studies were more variable. Some trials reported improvements in anxiety, and depressive symptoms, while others showed no significant differences compared with control groups. Studies that combined probiotics with antidepressants or exercise demonstrated the most pronounced reductions in anxiety and depression. Mechanistic insights from human studies partially aligned with animal evidence, with several trials showing reductions in inflammatory markers (IL-6, TNF-α), improvements in neuroendocrine measures (serotonin, dopamine, cortisol), stabilization of metabolic markers, and favorable shifts in gut microbiota, although these effects were not consistent across all studies. Probiotics appear to be safe within the intervention periods of the reviewed studies (<24 weeks), as no serious adverse effects were reported. Substantial heterogeneity across studies, including variations in cancer type, intervention duration, probiotic strains, formulations, dosages, and study design combined with small sample sizes, restricts the ability to draw definitive conclusions. Rigorously designed randomized controlled trials with larger sample sizes and mechanistic biomarkers are required to confirm the efficacy of probiotics for relieving anxiety and depression in the cancer population.

## 1. Introduction

As the population of cancer survivors grows, psychological distress, including anxiety, depressive symptoms, and depression, is increasingly recognized as a common concern that often begins at diagnosis and can persist throughout disease trajectory [1]. Depression occurs approximately two to three times more frequently among individuals with cancer compared to the general public [2], and 30% to 50% of individuals with cancer experience psychological distress such as anxiety and depressive symptoms [3,4]. Moreover, the risk of suicide increases substantially following a cancer diagnosis, particularly among individuals aged 15 to 39 years, who exhibit a 2.39-fold higher risk than their peers without cancer [5].

Multiple psychosocial and biological factors contribute to the development of anxiety and depression in cancer. Iatrogenic distress arising from poor communication and unmet psychological needs substantially contributes to these conditions [6]. Cancer treatments such as chemotherapy and radiotherapy can disrupt the gut microbiome, increase gut permeability, and trigger systemic inflammation [7,8]. These changes, in turn, influence key neurobiological pathways by dysregulating of the hypothalamic–pituitary–adrenal (HPA) axis and reducing levels of serotonin and brain-derived neurotrophic factor (BDNF), thereby increasing vulnerability to psychological distress [7,9,10,11,12].

Current management options for cancer-related anxiety and depression include pharmacological treatments (e.g., S-ketamine and Escitalopram), psychotherapeutic approaches (e.g., cognitive behavioral therapy (CBT), mindfulness-based therapies), complementary modalities (e.g., acupressure) and lifestyle interventions (e.g., dietary and exercise interventions) [7,13,14,15,16,17]. However, the effectiveness of these treatments varies and many patients experience adverse effects [3] or limited benefit [7,18,19,20,21,22,23,24]. These limitations have prompted growing interest in complementary strategies that target biological pathways implicated in cancer-related psychological distress, including the gut–brain axis.

Probiotics have emerged as a potential strategy to manage anxiety and depression by influencing the gut–brain axis. They can help restore microbiome balance, strengthen gut barrier integrity, reduce inflammation, and influence neurotransmitter pathways such as serotonin synthesis [7,17,23,24]. Evidence from animal models of cancer has shown that microbial interventions, such as Tibetan kefir fermented goat milk dominated by *Lactobacillus kefiranofaciens*, can reduce depression- and anxiety-like behaviors, suppress inflammation, rebalance stress hormone activity, and improve gut integrity and microbial diversity [3,25,26]. Recent human studies indicate that similar mechanisms are relevant for individuals with cancer. Clinical trials using probiotic strains such as *Lactobacillus rhamnosus*, *Lactobacillus helveticus*, *Bifidobacterium longum*, *Lactobacillus acidophilus*, and *Lactobacillus casei* have reported reductions in psychological distress and improvements in mood [7,27].

Despite increasing interest in microbiome-based interventions, evidence on the effects of probiotics on anxiety and depression in cancer remains limited and inconsistent, largely due to substantial variation in probiotic formulations, study designs, sample sizes, cancer types, and outcome measures. To our knowledge, no comprehensive review has provided an integrated, mechanistic synthesis of how probiotics may influence psychological distress in this population. Animal studies offer essential insight into biological pathways that cannot be directly examined in humans, while clinical studies help determine whether these mechanisms translate into meaningful patient outcomes. Therefore, this systematic review brings together both pre-clinical and clinical evidence to clarify the biological pathways through which probiotics/kefir may modulate anxiety and depressive symptoms in cancer and to evaluate their potential effects on related outcomes such as stress, sleep, fatigue, and quality of life.

## 2. Methods

### 2.1. Study Selection Criteria

In this systematic review, we included original research studies conducted in animal models or human populations. Human studies were limited to adults diagnosed with any cancer type or stage, including individuals undergoing active treatment and those who had completed treatment. To capture the full range of real-world cancer survivorship contexts, we did not exclude studies based on participants’ comorbid physical or mental health conditions. The interventions of interest were probiotics or kefir administered alone or in combination with other treatments. Primary outcomes included anxiety, depressive symptoms, or clinical depression. Eligible studies were published in English up to 21 May 2025. Exclusion criteria included reviews, book chapters, letters, editorials, theses or dissertations, secondary data analyses, conference abstracts, and case reports.

### 2.2. Target Database and Search Strategies

The online databases PubMed, Embase, CINAHL, Web of Science, and PsycINFO were systematically searched to identify studies evaluating the effect of probiotics on anxiety, depressive symptoms, and depression in both animal models and human studies with cancer. The key search terms included cancer, probiotics, anxiety, depression or depressive symptoms.

### 2.3. Review Process

Four reviewers (HC, TA, LZ, and AE) independently screened the titles and abstracts of all included articles to assess their relevance to the effect of probiotics on anxiety and depression in cancer-related animal and human studies. The same reviewers then independently evaluated the full-text articles. Any discrepancies during the screening or full-text review process were discussed and resolved by a fifth reviewer (ZAB), resulting in the final set of studies included in this systematic review.

### 2.4. Quality Appraisal

The risk of bias in the animal studies was evaluated using the SYRCLE risk of bias tool [28]. SYRCLE is an adaptation of the Cochrane Risk of Bias tool for pre-clinical research and evaluates domains such as sequence generation, allocation concealment, random housing, blinding, outcome assessment, incomplete data, selective reporting, and other potential sources of bias, with ratings of low, high, or unclear risk.

The risk of bias in the human studies was assessed using the revised Cochrane risk of bias for randomized trials (RoB2) tool, version 2019 [29]. RoB2 is specifically designed to evaluate randomized controlled trials (RCTs) across five key domains: the randomization process, deviations from intended interventions, missing outcome data, measurement of the outcome, and selection of the reported result with judgments of low risk, some concerns, or high risk of bias. RoB 2 is widely used in systematic reviews of randomized trials [30]. Two reviewers (HL and YL) independently conducted the assessments, and any discrepancies were reviewed and resolved by a third reviewer (ZAB).

### 2.5. Data Extraction and Synthesis

Information extracted from each study included the following: (1) study characteristics (e.g., authors, year of publication, country of origin, study design, and sample size); (2) participant details (e.g., demographic and clinical characteristics); (3) intervention characteristics (e.g., type, dose, and duration of probiotic treatment); (4) outcome (e.g., anxiety, depressive symptoms, and depression). Data extraction was independently conducted by four reviewers (HC, TA, LZ, and AE), and the accuracy of the extracted data was reviewed and confirmed by a fifth reviewer (ZAB).

## 3. Results

### 3.1. Literature Search

**Figure 1:**
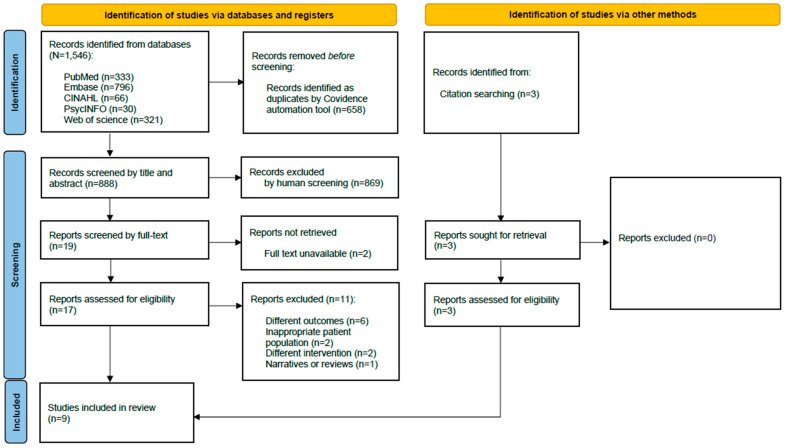
Flow Diagram of Study Selection.

An initial search identified a total of 1546 records across five databases, including PubMed (n = 333), Embase (n = 796), CINAHL (n = 66), PsycINFO (n = 30), and Web of Science (n = 321). The study selection process was conducted in compliance with the PRISMA guidelines. After removing duplicates (n = 658), 888 studies remained for screening, and three additional articles were found through manual reference checks and hand searches. Following title and abstract screening, 869 records were excluded for not meeting the inclusion criteria, leaving 19 articles for full-text review. Of these, two reports could not be retrieved or had no full text available, resulting in 17 full-text articles assessed for eligibility. Across all full-text articles assessed, 11 were excluded due to different outcomes (n = 6), inappropriate patient population (n = 2), different intervention (n = 2), or being narrative or systematic reviews (n = 1). A total of nine studies met all eligibility criteria and were included in the systematic review. The protocol for this systematic review was registered in PROSPERO (registration ID: 1080928), in accordance with PRISMA 2020 guidelines. The completed PRISMA 2020 checklist can be found in Supplementary Materials. The PRISMA flow diagram in Figure 1 provides a detailed overview of the search and study selection process.

### 3.2. Study Characteristics

A total of nine studies were included in this review. Five studies were conducted in China, one in Greece, one in Indonesia, one in Korea, and one in the United States. Of these, seven were conducted in humans, one in animals, and one included both humans and animals. Among the eight human studies, five were RCTs and three were non-randomized trials. The sample size of the included human studies varied from 24 to 266.

In terms of cancer types, one of the animal studies included a rat model of chemo-brain induced by doxorubicin administration and the other employed a mouse model of colorectal cancer (CRC) induced by azoxymethane (AOM) and 2% dextran sulfate sodium (DSS). The human studies involved individuals diagnosed with a range of cancers, including breast, laryngeal, gastric, bowel, rectal, pancreatic, colorectal, and liver cancer. Participant ages ranged from 18 to 85 years, and the proportion of female participants ranged from 33% to 100%, depending on cancer type.

Regarding probiotic interventions, studies in both animals and humans examined a wide variety of strains, formulations, and administration protocols. In animal studies, the probiotis intervention included a mixture of *Bifidobacterium longum*, *Lactobacillus acidophilus*, and *Enterococcus faecalis* (≥1.0 × 10^7^ CFU/210 mg), as well as Tibetan kefir goat milk containing *Lactobacillus kefiranofaciens* (*~83%*), administered daily for 120 days.

Across the included studies, probiotic interventions consisted of diverse bacterial formulations administered at varying doses, frequencies, and durations. Interventions using *Lactobacillus rhamnosus* Rosell-11 and *Lactobacillus helveticus* Rosell-52 delivered 2 × 10^9^ CFU per dose, taken twice daily for eight weeks. Another commonly used formulation contained *Lactobacillus rhamnosus* R0011 and *Lactobacillus acidophilus* R0052 at a dose of 2 × 10^9^ CFU, administered twice daily for periods ranging from six to twelve weeks. Multi-strain combinations were also represented, including preparations containing *Bifidobacterium longum*, *Lactobacillus acidophilus*, and *Enterococcus faecalis*, each provided at ≥1.0 × 10^7^ CFU per 210 mg and taken twice daily throughout the intervention period. Additional protocols included the administration of *Clostridium butyricum* at 420 mg per capsule, taken twice daily for two weeks, and a high-dose psychobiotic mixture consisting of *Bifidobacterium animalis* subsp. *lactis* BS01 (2.5 × 10^10^ CFU), *Bifidobacterium breve* BR03 (1 × 10^10^ CFU), *Bifidobacterium longum* BL04 (8 × 10^9^ CFU), and *Lacticaseibacillus rhamnosus* GG (4.5 × 10^10^ CFU), taken twice daily for 30 days. Other regimens included triple-viable *Bifidobacterium* capsules (0.21 g per dose) administered twice daily for eight weeks, as well as mixed probiotic cultures containing *Bifidobacterium*, *Enterococcus*, and *Lactobacillus acidophilus*, taken as two capsules three times daily for four weeks. A fermented milk beverage was also used as a probiotic source, providing 12 microbial strains and approximately 15–20 billion CFU per 8-oz serving, consumed three times weekly over a twelve-week period.

In terms of intervention duration, the most common probiotic intervention lasted 8 to 12 weeks, reflecting the typical length of controlled supplementation protocols. Fixed-duration regimens ranged more broadly from 2 to 12 weeks. However, in one study in which probiotics were administered throughout the entire course of chemotherapy, the intervention duration depended on each patient’s treatment schedule and could extend to approximately 24 weeks.

The biomarkers in animal studies were microbiota composition, blood metabolites, including, p-Mentha-1,8-dien-7-ol, hypothalamic–pituitary–adrenal activity measured by hypothalamic corticotropin-releasing factor (CRF), serum corticosterone (CORT), and hippocampal glucocorticoid receptor (GR) gene expression; inflammation measured by pro- and anti-inflammatory markers such as IL-6, IL-1β, TNF-α, IL-17, and IL-10 serum lipopolysaccharide (LPS); tryptophan metabolism measured by serum 5-hydroxytryptamine (5-HT), and colon 5-hydroxytryptophan (5-HTP) levels.

The biomarkers in human studies included inflammation- and immunity-related measures such as CRP, IL-6, IL-1β, TNF-α, procalcitonin (PCT), D-lactic acid, total monocyte counts, classical, intermediate, and nonclassical monocyte subsets, and cytokine production. Neurobiological and neuroendocrine markers included corticotropin-releasing factor (CRF), cortisol, serotonin, dopamine, oestradiol, and heart rate. Gut-related outcomes included microbiome diversity and composition, along with indicators of gut barrier integrity such as LPS. Nutritional biomarkers were evaluated using total protein (TP), prealbumin (PA), hemoglobin (HB), serum albumin (ALB), and total lymphocyte count (TLC). A summary of biomarker comparisons is provided in Table 1.

In animal studies behavioral outcomes includes depressive-like behaviors measured by forced swim test (FST), tail suspension test (TST), sucrose preference test (SPT), open field test, and elevated plus maze (EPM). In human studies, anxiety and depression as main outcomes were assessed with validated clinical instruments, including the Depression, Anxiety, and Stress Scale-42 (DASS-42), the Self-Rating Anxiety Scale (SAS), Self-Rating Depression Scale (SDS), Hamilton Anxiety Scale (HAMA), Hamilton Depression Scale (HAMD), Hamilton Depression Rating Scale (HDRS), Generalized Anxiety Disorder-7 (GAD-7), Patient Health Questionnaire-9 (PHQ-9), and Beck Depression Inventory (BDI and BDI-II). Other behavioral outcomes reported in human studies included incidence of CRCI via neuropsychological battery, fatigue, stress, quality of life, gastrointestinal symptoms (e.g., dyspepsia, dietary restriction, nausea, reflux, abdominal cramps, stomach fullness, flatulence, hiccups, and dysphagia), and sleep (e.g., difficulties with early, middle, and late-night awakenings as well as overall sleep quality). Study characteristics can be found in Table 2.

#### Risk of Bias

The two animal studies were evaluated using the SYRCLE risk of bias tool, which indicated a moderate to high risk of bias in both. The main concerns included insufficient reporting of sequence generation and allocation concealment, inadequate descriptions of blinding, small sample sizes, and potential selective outcome reporting (Table 3) [3,27]. For human studies, the quality was assessed using the RoB 2. Three trials were classified as low risk of bias due to robust randomization, blinding, and appropriate measures [7,17,31]. Four studies were identified as having some concerns, preliminary due to unclear or insufficiently reported randomization methods and blinding [27,32,33,34]. One study was rated as high risk due to non-random allocation [35].The most common methodological issues across studies were related to the randomization process and blinding (Table 4). To improve methodological transparency, we acknowledge that although two reviewers independently conducted all quality assessments, formal inter-rater reliability metrics (e.g., Kappa coefficient) were not calculated.

**Table 1:** Comparison of Similar Biomarkers Assessed in Human and Animal Studies of Probiotic and Kefir.

Category	Human Studies	Animal Studies
Neurobiological & Neuroendocrine Markers	• **Fitrikasari et al. (2024) [7]:** Serum serotonin ↑ in probiotic group (NS, *p* = 0.38); control also ↑ (NS, *p* = 0.09); post-intervention serotonin higher in control vs. probiotic (*p* = 0.01). • **Lu et al. (2025) [33]:** Dopamine ↑, serotonin ↑, cortisol ↓ in probiotic + mirtazapine vs. mirtazapine alone (*p* < 0.05). • **Yang et al. (2016) [32]:** Placebo group ↑ CRF and ↑ heart rate (*p* < 0.05); probiotic group stable. Between groups: probiotic group had ↓ CRF and ↓ heart rate (*p* < 0.05). • **Juan et al. (2022) [27]:** No significant changes in estradiol or cortisol.	• **Sun et al. (2024) [3]:** ↓ hypothalamic CRF (*p* < 0.001); ↓ serum CORT; ↑ hippocampal GR/Nr3c1 (*p* < 0.001); MR unchanged; kefir restored serum 5-HT and 5-HTP.
Systemic Inflammatory & Immune Markers	• **Smoak (2021) [35]:** No significant changes in serum IL-6 or CRP; whole-blood LPS-stimulated IL-6 ↓ over time (*p* = 0.019); no TNF-α effects. ↑ circulating monocytes (*p* = 0.035), ↑ classical monocytes (*p* < 0.001), ↓ nonclassical monocytes (*p* = 0.040), ↑ total monocytes (*p* = 0.041), ↓ intermediate monocytes (*p* = 0.027). • **Juan et al. (2022) [27]:** No significant IL-1β, IL-6, or TNF-α changes.	• **Sun et al. (2024) [3]:** ↓ hippocampal & serum IL-6, IL-1β, TNF-α, IL-17 (all *p* < 0.001); ↑ IL-10 (*p* < 0.001 or *p* < 0.01). T-cell transcriptional markers: ↑ T-bet; ↓ GATA3 and ↓ Ror-γT (all *p* < 0.001). Colon: ↓ IL-6, ↓ IL-1β, ↓ TNF-α, ↓ IL-17A; ↑ IL-10 (*p* < 0.001 or *p* < 0.01).
Gut Barrier Integrity & Microbial Translocation	• **Peng et al. (2021) [34]:** ↓ D-lactic acid, ↓ PCT, ↓ endotoxin in probiotic and probiotic + paroxetine groups (*p* < 0.05); greater reductions with the combination (*p* < 0.05). • **Smoak (2021) [35]:** Serum LPS time–group interaction significant (*p* = 0.01) favoring kefir.	• **Sun et al. (2024) [3]:** ↓ serum LPS (*p* < 0.001). ↑ Claudin-1, ↑ Occludin, ↑ ZO-1 (*p* < 0.001 or *p* < 0.01).
Gut Microbiota Composition & Diversity	• **Juan et al. (2022) [27]:** No α- or β-diversity differences; ↓ *Streptococcus* (*p* = 0.023), ↓ *Tyzzerella* (*p* = 0.033), ↑ *Enterococcus* after chemotherapy. • **Lu et al. (2025) [33]:** ↑ *L. acidophilus*, ↑ *Bifidobacterium*; ↓ *E. coli*, ↓ *E. faecalis*; all changes greater in combination therapy (*p* < 0.05).	• **Sun et al. (2024) [3]:** Kefir shifted diversity toward healthy controls; ↑ α- and β-diversity vs. CRC + CUMS. ↑ *Bifidobacterium*, ↑ *Dubosiella*, ↑ *Allobaculum*, ↑ *Ileibacterium*; ↓ *Desulfovibrio*. • **Juan et al. (2022) [27]:** Probiotics ↑ *Enterococcus*, *Bifidobacterium*, and *Helicobacter*; reversed chemotherapy-induced α- and β-diversity disruption.
Metabolites & Nutritional Outcomes	• **Juan et al. (2022) [27]:** ↓ glucose (*p* = 0.02), ↓ LDL (*p* = 0.03); nine metabolites differed (e.g., p-Mentha-1,8-dien-7-ol, linoelaidyl carnitine, phenylalanyl-tryptophan). • **Lu et al. (2025) [33]:** ↑ Hb, ↑ albumin, ↑ prealbumin—all greater with probiotics + mirtazapine (*p* < 0.001). • **Peng et al. (2021) [34]:** ↑ TP, ↑ ALB, ↑ Hb, ↑ PA, ↑ TLC in both groups (*p* < 0.05); greater increases in combo group (*p* < 0.05).	• **Juan et al. (2022) [27]:** ↑ plasma p-Mentha-1,8-dien-7-ol in saline-treated (*p* = 0.0086) and chemotherapy-treated rats (*p* = 0.0017).
Oxidative Stress & Neural Plasticity	• No human studies reported oxidative stress or synaptic plasticity markers.	• **Juan et al. (2022) [27]:** ↓ hippocampal ROS (*p* < 0.05); ↓ Iba-1 (*p* = 0.0004); ↓ GFAP (*p* = 0.0012). ↑ LTP, ↑ PSD95, ↑ synaptophysin (all *p* < 0.001).

↓ indicates a decrease; ↑ indicates an increase. LTP: long-term potentiation, ROS: reactive oxygen species, CRF: corticotropin-releasing factor, CORT: corticosterone, 5-HT: 5-hydroxytryptamine (serotonin), 5-HTP: 5-hydroxytryptophan, LDL: low-density lipoprotein cholesterol, LPS: lipopolysaccharide, PCT: procalcitonin, TP: total protein, Hb: hemoglobin, PA: prealbumin, ALB: serum albumin, TLC: total lymphocyte count, IL-1β: interleukin-1 beta, IL-6: interleukin-6, IL-10: interleukin-10, IL-17/IL-17A: interleukin-17A, TNF-α: tumor necrosis factor-alpha, CRP: C-reactive protein, Iba-1: ionized calcium-binding adapter molecule 1, GFAP: glial fibrillary acidic protein, T-bet: T-box transcription factor TBX21, GATA3: GATA-binding protein 3, Ror-γT: retinoid-related orphan receptor gamma T, ZO-1: zonula occludens-1.

**Table 2:** Characteristics of Studies Investigating Probiotics for Anxiety and Depression in Cancer.

First author/ Year/Country	Population/Cancer Type	Probiotic Strain(s) /Dosage/Duration	Interventions	Outcomes	Behavioral Findings	Biomarkers Findings
**Juan et al.** **/2022/China** **[27]**	Animals/Rat model of chemotherapy-related cognitive impairment (CRCI) model	*Bifidobacterium longum*, *Lactobacillus acidophilus*, *Enterococcus faecalis* (≥1.0 × 10^7^ CFU/210 mg)/NR	Saline + Vehicle (Control); Saline + Probiotic; Chemotherapy + Vehicle (Chemo-brain model); Chemotherapy + Probiotic	Hippocampal LTP, synaptic plasticity (PSD95, synaptophysin), ROS and oxidative stress, glial activation (Iba-1, GFAP), plasma metabolites (including p-Mentha-1,8-dien-7-ol), gut microbiome diversity and composition (16S rRNA sequencing)	NR	**Probiotics Biomarker Outcomes:** ↑ plasma p-Mentha-1,8-dien-7-ol (*p* = 0.0086 and *p* = 0.0017) Reversal of chemotherapy-induced microbiome α/β dysbiosis (*p* < 0.01) ↑ Enterococcus, ↑ Bifidobacterium, ↑ Helicobacter, **MDO Injection Mechanistic Biomarker Outcomes:** Restored LTP (*p* < 0.001) ↑ PSD95 (*p* < 0.0001) Synaptophysin unchanged (*p* = 0.604) ↑ PSD95 + synaptophysin colocalization (*p* < 0.0001) ↓ ROS (*p* < 0.0001) ↓ Iba1 (*p* = 0.0004) ↓ GFAP (*p* = 0.0012)
**Sun et al./2024/** **China** **[3]**	Animals/Colorectal cancer (CRC) induced by AOM + DSS with CUMS	Tibetan kefir goat milk (community dominated by *Lactobacillus kefiranofaciens*)/once daily for 120 days	CRC + CUMS; CRC + CUMS + Fluoxetine; CRC + CUMS + Tibetan kefir goat milk; CRC only; control group	Depressive- and anxiety-like behavior (FST, TST, SPT, open-field test, EPM), hypothalamic CRF, serum CORT, serum LPS, inflammatory cytokines (TNF-α, IL-1β, IL-6, IL-17) in serum/hippocampus/colon, serum 5-HT and colonic 5-HTP, hippocampal GR (Nr3c1) and MR (Nr3c2) expression, colonic tight junction markers (Claudin-1, ZO-1), and gut microbiome diversity/composition (16S rRNA sequencing)	**CRC + CUMS vs. Control:** ↑ immobility (FST/TST; *p* < 0.001), ↓ open-arm time (EPM; *p* < 0.001); **CRC + CUMS vs. CRC:** ↑ despair-like behavior (FST *p* < 0.001, TST *p* < 0.05); **Kefir vs. CRC + CUMS:** ↓ immobility (FST/TST; *p* < 0.001 or *p* < 0.05), ↑ sucrose preference/↓ anhedonia (SPT; *p* < 0.001), ↓ anxiety-like behavior (open-field/EPM; *p* < 0.001); **Fluoxetine vs. CRC + CUMS:** ↓ immobility (FST/TST; *p* < 0.001 or *p* < 0.05), ↑ sucrose preference/↓ anxiety-like behavior	**CRC + CUMS (vs. control):** ↑ serum & hippocampal cytokines: IL-6, IL-1β, TNF-α, IL-17 (*p* < 0.001) ↑ colonic pro-inflammatory cytokines: IL-6, IL-1β, TNF-α, IL-17A (*p* < 0.001) and ↓ IL-10 (*p* < 0.001) ↑ serum LPS (*p* < 0.001) ↓ tight junction proteins in colon: Claudin-1, ZO-1, Occludin (*p* < 0.001) ↓ serum 5-HT and ↓ colonic 5-HTP (*p* < 0.001), ↓ gut microbiome α-diversity; β-diversity separated from controls (PCoA discrete), *↑ Firmicutes, ↓ Bacteroidetes → ↑ Firmicutes:Bacteroidetes* ratio; Genus shifts: ↑ *Lactobacillus, Turicibacter, Desulfovibrio; ↓ Ileibacterium, Muribaculaceae, Clostridia, Lachnospiraceae*; **Kefir group (vs. CRC + CUMS):** ↓ hypothalamic CRF (*p* < 0.001) and ↓ serum CORT,↑ hippocampal GR/Nr3c1 (*p* < 0.001), ↓/inhibited serum & hippocampal cytokines: IL-6, IL-1β, TNF-α, IL-17, Alleviated colonic inflammation imbalance (including ↑ IL-10) (*p* < 0.001 or *p* < 0.01) ↓ serum LPS, ↑ tight junction proteins: Claudin-1, ZO-1, Occludin (*p* < 0.001 or *p* < 0.01) ↑/restored serum 5-HT and colonic 5-HTP, Microbiome (implied vs. CRC + CUMS): ↑ *Dubosiella, Bifidobacterium, Ileibacterium, Allobaculum and ↓ Lactobacillus, Bacillus, Desulfovibrio, Streptococcus*; **Fluoxetine group (vs. CRC + CUMS):** ↓ hypothalamic CRF (*p* < 0.001) and ↓ serum CORT, ↑ hippocampal GR/Nr3c1 (*p* < 0.001), ↓/inhibited serum & hippocampal cytokines: IL-6, IL-1β, TNF-α, IL-17, alleviated colonic inflammation imbalance (including ↑ IL-10) (*p* < 0.001 or *p* < 0.01), ↓ serum LPS (described as reduced), ↑ tight junction proteins: Claudin-1, ZO-1, Occludin (*p* < 0.001 or *p* < 0.01) ↑/restored serum 5-HT and colonic 5-HTP, Microbiome: effect at the phylum level was described as greater than kefir
**Fitrikasari et al./2024/** **Indonesia** **[7]**	Humans/Mixed cancer types undergoing chemotherapy	*Lactobacillus rhamnosus* Rosell-11 and *Lactobacillus helveticus* Rosell-52/2 × 10^9^ CFU, twice daily, 8 weeks	Probiotics vs. Placebo	Depression, anxiety, and stress (DASS-42), serum serotonin	**Within probiotic group:** non-significant reductions in depression (*p* = 0.32), anxiety (*p* = 0.91), and stress (*p* = 0.58), but significant ↓ total DASS-42 (*p* = 0.001). **Within placebo group:** significant ↓ total DASS-42 (*p* = 0.002), ↓ depression (*p* = 0.01), ↓ anxiety (*p* = 0.02), ↓ stress (*p* = 0.007). **Between groups:** no significant differences in depression, anxiety, or stress scores; total DASS-42 significantly lower in probiotic vs. placebo (*p* = 0.048)	**Within probiotic group: serum serotonin ↑** (NS; *p* = 0.38). **Within placebo group:** serum serotonin ↑ (NS; *p* = 0.09). **Between groups:** post-intervention serum serotonin higher in placebo vs. probiotic (*p* = 0.01)
**Juan et al./2022/** **China** **[27]**	Humans/Breast cancer	*Bifidobacterium longum* (≥1.0 × 107 CFU/210mg), *Lactobacillus acidophilus* (≥1.0 × 107 CFU/210mg) and *Enterococcus faecalis* (≥1.0 × 107 CFU/210mg)/twice daily, ~12–24 weeks (throughout chemotherapy)	Probiotics vs. Placebo	CRCI incidence (HVLT-R, BVMT-R, JoLO, VFT, DS, DST), anxiety & depression (SAS, SDS), biochemical & metabolic markers (GLU, LDL, IL-1β, IL-6, TNF-α, oestradiol, cortisol), gut microbiota composition (16S rRNA sequencing)	**Between groups:** ↓ total CRCI incidence (35% vs. 81%; *p* < 0.001); ↓ mild CRCI (*p* = 0.003); ↓ moderate CRCI (*p* < 0.001); ↑ cognitive performance on HVLT-R IR (*p* = 0.003), HVLT-R DR (*p* = 0.001), BVMT-R IR (*p* = 0.001), BVMT-R DR (*p* = 0.003), BVMT-R IF (*p* < 0.001), JoLO (*p* = 0.007), VFT (*p* < 0.001); no significant differences in anxiety or depression	**Between groups:** ↓ blood glucose (*p* = 0.02), ↓ LDL (*p* = 0.03) in probiotic vs. placebo at chemotherapy completion; no significant group differences in IL-1β, IL-6, TNF-α, estradiol, or cortisol. Gut microbiota: ↑ *Enterococcus* (*p* < 0.001), ↓ *Streptococcus*(*p* = 0.023), ↓ *Tyzzerella_3* (*p* = 0.033) in probiotics; Between groups: significant differences in metabolite change for Linoelaidyl carnitine (*p* = 0.048), Glycylproline (*p* = 0.01), p-Mentha-1,8-dien-7-ol (*p* = 0.006), Carnosol (*p* = 0.009), 3,4,5-Trimethoxyphenyl glucoside (*p* = 0.007), 1,3-diazinane-2,4-dione (*p* = 0.02), Adamantan-1-amine (*p* = 0.02), Phenylalanyl-Tryptophan (*p* = 0.03), and 1-aminocyclopropane-1-carboxylic acid (*p* = 0.02)
**Yang et al./2016/** **China** **[32]**	Humans/Laryngeal cancer (pre-surgery)	*Clostridium butyricum*, 420 mg per capsule/twice daily, 2 weeks	Probiotics vs. Placebo	Anxiety (HAMA), serum CRF, heart rate	**Within probiotic group:** ↓ HAMA scores before surgery (*p* < 0.05). **Within placebo group:** ↑ HAMA score. **Between groups:** lower pre-surgery HAMA in probiotic vs. placebo (*p* < 0.05)	**Within probiotic group:** CRF and heart rate remained stable pre-surgery. **Within placebo group:** ↑ serum CRF and ↑ heart rate before surgery (*p* < 0.05). **Between groups:** lower serum CRF (*p* < 0.05) and lower heart rate (*p* < 0.05) in probiotic vs. placebo
**Tzikos et al./2025/** **Greece** **[17]**	Humans/Gastrointestinal cancers (gastric, bowel, rectal, pancreatic)	*Bifidobacterium animalis* subsp. *lactis* LMG P-21384 [BS01] [2.50 × 1010 cfu/dose], *Bifidobacterium breve* DSM 16604 [BR03] [1.00 × 1010 cfu/dose], *Bifidobacterium longum* DSM 16603 [BL04] [8.00 × 109 cfu/dose], and *Lacticaseibacillus rhamnosus* ATCC 53103 [GG] [4.50 × 1010 cfu/sachet twice daily, 4 weeks (minimum viable count ≥1 × 10^9^ CFU at shelf-life end)	Probiotics vs. Placebo	Depression and anxiety (BDI-II, HDRS, GAD-7), stress (PSS-14), [3] quality (BDI-II [Q16] and HDRS [questions Q4, Q5, Q6])	**Within probiotic group:** ↓ HDRS depression scores and ↓ proportion of depressed participants (*p* < 0.001); ↓ GAD-7 anxiety (*p* = 0.009 at T2; *p* = 0.002 at T3); ↓ perceived stress (PSS-14) (*p* = 0.001 to *p* < 0.001); ↑ proportion of non-depressed, non-anxious, and low-stress individuals; Improved sleep quality (significant improvements across HDRS/BDI-II sleep items). **Within placebo group:** ↑ depression (HDRS) (*p* = 0.002 to *p* < 0.001); ↑ anxiety (GAD-7) (*p* < 0.001); ↑ perceived stress (PSS-14) (*p* = 0.043 to *p* < 0.001); Deterioration in sleep measures. **Between groups:** ↓ risk of depression in probiotic vs. placebo (T2 RR = 0.18; T3 RR = 0.10; both *p* < 0.001); Lower risk of anxiety (T2 RR = 0.30; T3 RR = 0.13; *p* < 0.001); Greater reduction in stress (*p* = 0.016 at T2; *p* < 0.001 at T3); Better sleep quality in probiotic vs. placebo (significant across sleep-related HDRS/BDI-II items)	NR
**Lee et al./2014/** **Republic of Korea** **[31]**	Humans/Colorectal cancer survivors	Lacidofil: *L. rhamnosus* R0011 + *L. acidophilus* R0052 (2 × 10^9^ CFU/capsule)/twice daily, 12 weeks	Probiotics vs. Placebo	Depression/anxiety (PHQ-9), CRC-related QoL (FACT-G, FACT-C), fatigue (FACT-F), neurologic symptoms (FACT-NTX), bowel symptoms (ROME III)	**Within probiotic group:** ↓ PHQ-9 (*p* = 0.01), ↓ IBS-like bowel symptoms (*p* = 0.03), ↑ FACT-C (*p* = 0.04), ↑ FACT-F (*p* = 0.02). **Within placebo:** no significant PHQ-9 change (*p* = 0.33). **Between groups:** ↓ bowel symptoms (*p* = 0.03), ↑ functional well-being (FWB; *p* = 0.04), ↑ FACT-C (*p* = 0.04) favoring probiotics.	NR
**Lu et al./2025/** **China** **[33]**	Humans/Gastric cancer with depression after radical resection	Bifidobacterium triple viable bacteria capsules, 0.21 g/twice daily, 8 weeks	Probiotics + mirtazapine vs. mirtazapine alone	Depression (SDS, HAMD, BDI), QoL (European Organization for Cancer Research and Treatment of the Core Scale of Life Gastric Cancer Scale), neuroendocrine markers (dopamine, serotonin, cortisol), nutritional status (Hb, ALB, PA), intestinal flora (colony counts)	**Within probiotic + mirtazapine group:** ↓ SDS, ↓ HAMD, ↓ BDI (all *p* < 0.05); improved QoL domains (dysphagia, stomach pain, dietary restriction, hiccups, anxiety, total symptom burden; all *p* < 0.05). **Between groups:** greater reductions in SDS, HAMD, BDI and QoL symptom scores in probiotic + mirtazapine vs. mirtazapine alone (all *p* < 0.001)	**Within probiotic + mirtazapine group:** ↑ dopamine, ↑ serotonin, ↓ cortisol (all *p* < 0.05); ↑ *Lactobacillus acidophilus* and *Bifidobacterium*, ↓ *Escherichia coli* and *Enterococcus faecalis* (all *p* < 0.05); ↑ hemoglobin, ↑ serum albumin, ↑ prealbumin (all *p* < 0.05). **Within mirtazapine-only group:** same directions of change (↑ dopamine/serotonin, ↓ cortisol; ↑ Lactobacillus/Bifidobacterium, ↓ *E. coli*/*E. faecalis*; ↑ Hb/ALB/PA), but smaller improvements (all *p* < 0.05). **Between groups:** greater ↑ dopamine and serotonin and greater ↓ cortisol (all *p* < 0.001), larger ↑ *L. acidophilus* and *Bifidobacterium* and larger ↓ *E. coli* and *E. faecalis* (all *p* < 0.001), and greater ↑ Hb, ALB, PA (all *p* < 0.001)
**Peng et al./2021/** **China** **[34]**	Humans/Liver cancer with type 2 diabetes mellitus and GI dysfunction	Capsules containing *Bifidobacterium*, *Enterococcus,* and *Lactobacillus acidophilus*/2 capsules, 3× daily, 4 weeks	Probiotics + paroxetine vs. Probiotics alone	Anxiety and depression (HAMA, HAMD), gut barrier markers (D-lactic acid, PCT, endotoxin), nutritional status (TP, ALB, Hb, PA, TLC)	**Within probiotic + paroxetine group:** ↓ HAMA and ↓ HAMD (both *p* < 0.05). **Within probiotics-only group:** ↓ HAMA and ↓ HAMD (both *p* < 0.05). **Between groups:** greater reductions in HAMA and HAMD in probiotic + paroxetine vs. probiotics alone (both *p* < 0.001)	**Within probiotic + paroxetine group:** ↓ D-lactic acid, ↓ PCT, ↓ endotoxin (all *p* < 0.05); ↑ TP, ↑ ALB, ↑ Hb, ↑ PA, ↑ TLC (all *p* < 0.05). **Within probiotics-only group:** similar directional changes (all *p* < 0.05). **Between groups:** greater ↓ D-lactic acid, ↓ PCT, ↓ endotoxin and greater ↑ TP, ALB, Hb, PA, TLC in probiotic + paroxetine vs. probiotics alone (all *p* < 0.05)
**Smoak et al./2021/** **USA** **[35]**	Humans/Cancer survivors in an exercise program	Kefir containing 12 probiotic strains (***Lactobacillus lactis***, ***Lactobacillus rhamnosus***, ***Streptococcus diacetylactis***, ***Lactobacillus plantarum***, ***Lactobacillus casei***, ***Saccharomyces florentinus***, ***Leuconostoc cremoris***, ***Bifidobacterium longum***, ***Bifidobacterium breve***, ***Lactobacillus acidophilus***, ***Bifidobacterium lactis***, and ***Lactobacillus reuteri***), ~15–20 billion CFU per 8 oz serving/3 times per week, 12 weeks	Kefir + exercise vs. Exercise alone	Depression (BDI), fatigue (Revised Piper Fatigue Scale), QOL (Ferrans and Powers Quality of Life Index III), gastric distress, serum LPS, plasma IL-6, CRP, monocyte subsets (total, classical CD14^+^CD16^−^; intermediate CD14^+^CD16^++^; nonclassical CD14^++^CD16^+^), whole blood LPS-stimulated IL-6 and TNF-α production	**Within kefir + exercise (KEF):** ↓ BDI: 51.4%, ↓ Fatigue: 39.3%, ↓ Gastric distress: 64.7%, **Within exercise-only control (CON):** ↓ BDI: 3.4%, ↓ Fatigue: 5.1%, ↓ Gastric distress: 23.6%, **Between groups (KEF vs. CON; time×group interaction):** KEF showed greater ↓ BDI (*p* = 0.046), greater ↓ Fatigue (*p* = 0.03), greater ↓ Gastric distress (*p* = 0.021), **Overall (main effect of time, all participants):** ↓ BDI (*p* = 0.032), ↓ Fatigue (*p* = 0.017), ↓ Gastric distress (*p* = 0.013)	**Within kefir + exercise (KEF):** ↓ Serum LPS: 35.4%, ↑ % immune cells that were monocytes: 47.3%, ↑ % classical monocytes: 18.0%, ↓ % nonclassical monocytes: 22.3%, **Within exercise-only control (CON):** ↑ Serum LPS: 13.6%, ↑ % immune cells that were monocytes: 3.8%, ↓ % classical monocytes: 4.4%, ↑ % nonclassical monocytes: 30.4%, **Between groups (KEF vs. CON; time×group interaction):** KEF showed greater ↓ Serum LPS (*p* = 0.01), greater ↑ % immune cells that were monocytes (*p* = 0.035), greater ↑ % classical monocytes (*p* < 0.001), greater ↓ % nonclassical monocytes (*p* = 0.040), **Overall (main effect of time, all participants):** ↑ total monocyte count (*p* = 0.041), ↓ % intermediate monocytes (*p* = 0.027), ↓ whole blood LPS-stimulated IL-6 production per monocyte (*p* = 0.019), ↓ whole blood LPS-stimulated TNF-α total production (*p* = 0.022), **No main effects or interactions:** resting/fasted serum CRP, resting/fasted serum IL-6, whole blood LPS-stimulated IL-6 total production (stim–unstim), TNF-α production per monocyte

↓ indicates a decrease; ↑ indicates an increase. NR: not reported, LTP: long-term potentiation, ROS: reactive oxygen species, AOM: azoxymethane, DSS: dextran sulfate sodium, CRF: corticotropin-releasing factor, CORT: corticosterone, GR: glucocorticoid receptor, MR: mineralocorticoid receptor, 5-HT: 5-hydroxytryptamine (serotonin), 5-HTP: 5-hydroxytryptophan, GLU: blood glucose, LDL: low-density lipoprotein, LPS: lipopolysaccharide, PCT: procalcitonin, TP: total protein, Hb: hemoglobin, PA: prealbumin, ALB: serum albumin, TLC: total lymphocyte count, IL: interleukin, TNF-α: tumor necrosis factor-alpha, GFAP: glial fibrillary acidic protein, Iba-1: ionized calcium-binding adapter molecule 1, CRCI: cancer-related cognitive impairment, HVLT-R: Hopkins Verbal Learning Test–Revised, BVMT-R: Brief Visuospatial Memory Test–Revised, JoLO: Judgment of Line Orientation test, VFT: Verbal Fluency Test, DS: Digit Span, DST: Digit Symbol Test, FST: forced swim test, TST: tail suspension test, SPT: sucrose preference test, EPM: elevated plus maze, DAI: disease activity index, CFU: colony-forming units, BDI: Beck Depression Inventory, BDI-II: Beck Depression Inventory-II, HDRS: Hamilton Depression Rating Scale, HAMD: Hamilton Depression Scale, GAD-7: Generalized Anxiety Disorder-7, SDS: Self-Rating Depression Scale, SAS: Self-Rating Anxiety Scale, PSS-14: Perceived Stress Scale-14, FACT-C: Functional Assessment of Cancer Therapy–Colorectal, FACT-F: Functional Assessment of Cancer Therapy–Fatigue, PHQ-9: Patient Health Questionnaire–9, QoL: quality of life.

**Table 3:** Risk of Bias Assessment of Included Animal Studies using SYRCLE risk of bias.

Domain	Sun et al./2024 [3]	Juan et al./2022 [27]
Sequence generation	Some concerns	Some concerns
Baseline characteristics	Low risk	Some concerns
Allocation concealment	Some concerns	Some concerns
Random housing	Some concerns	Some concerns
Blinding (caregivers/researchers)	Some concerns	Some concerns
Random outcome assessment	Some concerns	Some concerns
Blinding (outcome assessors)	Some concerns	Some concerns
Incomplete outcome data	Low risk	Low risk
Selective outcome reporting	Some concerns	Some concerns
Other sources of bias	Low risk	High risk
Overall risk of bias	Moderate to high	Moderate to high

**Table 4:** Risk of Bias (RoB 2) Assessment of Included Human Studies.

Study	Randomization Process	Deviations from Intended Interventions	Missing Outcome Data	Measurement of Outcomes	Selection of Reported Results	Overall Risk of Bias
**Fitrikasari et al./2024 [7]**	Low risk	Low risk	Low risk	Low risk	Low risk	Low risk
**Lee et al./2014 [31]**	Low risk	Low risk	Low risk	Low risk	Low risk	Low risk
**Tzikos et al./2025 [17]**	Low risk	Low risk	Low risk	Low risk	Low risk	Low risk
**Yang et al./2016 [32]**	Some concerns	Low risk	Low risk	Some concerns (self-reported scales)	Low risk	Some concerns
**Juan et al./2022 [27]**	Some concerns	Low risk	Low risk	Some concerns (self-reported scales)	Low risk	Some concerns
**Lu et al./2025 [33]**	Some concerns	Low risk	Low risk	Some concerns	Low risk	Some concerns
**Peng et al./2021 [34]**	Some concerns	Low risk	Low risk	Some concerns	Low risk	Some concerns
**Smoak et al./2021 [35]**	High risk (nonrandom allocation)	High risk (no placebo control)	Low risk	Some concerns	Some concerns	High risk

### 3.3. Impact of Probiotics on Biomarkers/Metabolic/Gut Microbiome

Multiple studies assessed diverse biomarkers to determine the effects of probiotics. Neurobiological/neurotransmitters and neuroendocrine markers indicate potential modulation. In a pre-clinical study, Sun et al. (2024) [3] reported that Tibetan kefir goat milk significantly ameliorated HPA axis dysregulation in CRC + CUMS mice. Compared with the CRC + CUMS group, the kefir group showed a marked reduction in hypothalamic CRF (*p* < 0.001) and diminished serum CORT. Kefir also significantly increased hippocampal glucocorticoid receptor (GR/Nr3c1) expression (*p* < 0.001), while mineralocorticoid receptor (MR/Nr3c2) expression was not significantly altered. In addition, kefir restored serum 5-HT and colonic 5-HTP [3]. Human studies have yielded conflicting findings regarding the impact of probiotics on neurobiological and neuroendocrine markers. Fitrikasari et al. (2024) [7] reported improved serum serotonin levels within the intervention group, though this change was not statistically significant (*p* = 0.38). The control group also exhibited increased serotonin levels without reaching significance (*p* = 0.09). Post-intervention, the control group had significantly higher serotonin levels than the intervention group (*p* = 0.01) [7]. Lu et al. (2025) [33] observed that within the probiotic plus mirtazapine group, dopamine and serotonin levels increased while cortisol levels decreased significantly after treatment (all *p* < 0.05). The mirtazapine-only group showed changes in the same direction, with increased dopamine and serotonin and reduced cortisol, though the magnitude of change was smaller (all *p* < 0.05). Between-group comparisons demonstrated significantly greater increases in dopamine and serotonin and a greater reduction in cortisol in the probiotic plus mirtazapine group compared with mirtazapine alone (all *p* < 0.001) [33].Yang et al. (2016) [32] found that, prior to surgery, CRF and heart rate remained stable in the probiotics group, while both increased significantly in the placebo group (*p* < 0.05). Between-group comparisons showed lower serum CRF and heart rate in the probiotics group (both *p* < 0.05) [32]. In contrast, Juan et al. (2022) found no significant differences in plasma oestradiol or cortisol levels between groups in breast cancer patients [27].

Regarding systemic inflammatory markers and immunity system related measures, Sun et al. (2024) [3] in a pre-clinical study showed that kefir markedly reduced hippocampal and serum IL-6, IL-1β, TNF-α, and IL-17 levels compared with the CRC + CUMS group (CRC + CUMS elevations vs. controls were reported at *p* < 0.001). In this study, Kefir also modulated T helper cell–related transcriptional markers in the hippocampus. Specifically, compared with the CRC group, the CRC + CUMS group showed down-regulation of T-bet gene expression and up-regulation of GATA3 gene expression, indicating an imbalance of Th1/Th2. In addition, compared with the blank control group, Ror-γT gene expression was upregulated, and both the kefir and fluoxetine groups significantly reversed this trend (*p* < 0.001). In the colon, kefir significantly decreased pro-inflammatory cytokines (IL-6, IL-1β, TNF-α, IL-17A) and increased IL-10 relative to CRC + CUMS (*p* < 0.001 or *p* < 0.01) [3]. In human studies, results are conflicting. While Smoak et al. (2021) [35] reported no significant effects for resting/fasted serum IL-6 or CRP (no changes over time or between groups), whole-blood LPS-stimulated IL-6 production per monocyte decreased significantly across all participants (*p* = 0.019), reflecting a main effect of time with no group interaction. LPS-stimulated TNF-α production per monocyte showed no significant main effects or interactions. Immune biomarkers also demonstrated modulation, with significant shifts in monocyte subsets: the exercise + kefir group showed greater increase in circulating monocytes (*p* = 0.035) and increase in classical monocytes (*p* < 0.001), along with greater reduction in nonclassical monocytes (*p* = 0.040) versus exercise alone. Additionally, total monocyte counts increase over time across groups (*p* = 0.041) and intermediate monocytes decrease over time (*p* = 0.027) [35]. In contrast to these promising results, Juan et al. (2022) found no significant changes in IL-1β, IL-6, or TNF-α within or between groups following probiotic implementation in breast cancer patients [27].

Markers of microbial translocation and gut barrier integrity, such as serum LPS, endotoxin, PCT, and D-lactic acid, decreased following probiotic interventions, indicating improved gut permeability. Peng et al. (2021) [34] reported that both the probiotics-only and probiotics plus paroxetine groups showed significant reductions in D-lactic acid, PCT, and endotoxin (*p* < 0.05). The group receiving both probiotics and paroxetine had significantly lower levels of D-lactic acid, PCT, and endotoxin compared to the probiotics only group (*p* < 0.05) [34]. Smoak et al. (2021) also demonstrated a significant time × group interaction for serum LPS (*p* = 0.01), with serum LPS decreasing in the kefir + exercise group (35.4%) and increasing in the exercise-only control group (13.6%) [35]. Similarly, in a pre-clinical study, Sun et al. (2024) reported that the kefir group reduced LPS concentration, suggesting a protective effect on the intestinal barrier [3]. In addition, significantly enhanced gene and protein expression of tight junction proteins, including, Claudin-1, Occludin, and ZO-1 (*p* < 0.001 or *p* < 0.01) relative to CRC + CUMS [3].

In terms of metabolites and nutritional outcomes, Juan et al. (2022) reported that in breast cancer patients, the probiotic group had significantly lower blood glucose (*p* = 0.02) and LDL levels (*p* = 0.03) than the placebo group at chemotherapy completion, while no differences were observed at baseline or mid-treatment. No other hematological measures differed between groups throughout chemotherapy [27]. In addition, nine serum metabolites showed significant intergroup differences across chemotherapy, involving pathways related to terpenoid and polyketide metabolism, pyrimidine metabolism, amino acid metabolism, and pantothenate/CoA biosynthesis. Metabolites that were significantly associated with group differences included Linoelaidyl carnitine (*p* = 0.048), Glycylproline (*p* = 0.01), *p*-Mentha-1,8-dien-7-ol (*p* = 0.006), Carnosol (*p* = 0.009), 3,4,5-Trimethoxyphenyl glucoside (*p* = 0.007), 1,3-diazinane-2,4-dione (*p* = 0.02), Adamantan-1-amine (*p* = 0.02), Phenylalanyl-Tryptophan (*p* = 0.03), and 1-aminocyclopropane-1-carboxylic acid (*p* = 0.02) [27]. In the corresponding animal model, probiotics induced parallel metabolite changes. Probiotic supplementation significantly increased plasma levels of *p*-Mentha-1,8-dien-7-ol both in saline-treated rats (*p* = 0.0086) and in chemotherapy-exposed rats (*p* = 0.0017) [27]. Among studies that combined probiotics with other interventions, Lu et al. (2025) [33] reported that after treatment, both the mirtazapine + probiotic group and the mirtazapine group showed increases in Hb, serum ALB, and PA levels. The mirtazapine + probiotic group exhibited significantly greater improvements in nutritional markers compared with mirtazapine alone, including larger increases in Hb (*p* < 0.001), serum ALB (*p* < 0.001), and PA (*p* < 0.001) [33]. Peng et al. (2021) [34] also reported that within the probiotic plus paroxetine group, TP, ALB, Hb, PA, and TLC significantly increased (*p* < 0.05) after four weeks. Similarly, within the probiotic-only group, TP, ALB, Hb, PA, and TLC increased (*p* < 0.05). However, the probiotic plus paroxetine group showed greater improvements, with greater increases in TP, ALB, Hb, PA, and TLC (*p* < 0.05) [34].

Microbiome analyses revealed favorable shifts in gut microbial composition, but findings are not conclusive. While Juan et al. (2022, human study) [27] reported no significant difference in Alpha-diversity or Beta-diversity between probiotics and placebo groups in breast cancer patients, a lower abundance of *Streptococcus* (*p* = 0.023), *Tyzzerella* (*p* = 0.033), and a higher abundance of *Enterococcus* was observed in probiotics group compared to the placebo after chemotherapy. In the corresponding animal model, chemotherapy produced marked alterations in Alpha- and Beta-diversity in the rat gut microbiome, and probiotic supplementation reversed this shift. At the genus level, probiotics increased the relative abundance of *Enterococcus*, *Bifidobacterium*, and *Helicobacter* in rats with or without chemotherapy [27]. Sun et al. (2024) [3] in a per-clinical study reported that kefir modulated gut microbial diversity and composition in mice, with the kefir group displaying a microbial community structure more similar to healthy controls than to CRC + CUMS. At the genus level, kefir increased beneficial taxa, including *Bifidobacterium, Dubosiella*, *Allobaculum*, and *Ileibacterium*, while reducing Desulfovibrio relative to CRC + CUMS. These shifts reflect improvements in microbial taxa linked to metabolic regulation, intestinal immunity, and lower inflammatory activity, indicating that kefir helped restore a healthier gut microbiota profile under conditions of chronic stress and colorectal cancer [3]. Lu et al. (2025) [33] reported that after treatment, both the mirtazapine plus probiotic group and the mirtazapine-alone group showed increases in *Lactobacillus acidophilus* and *Bifidobacterium*, along with reductions in *Escherichia coli* and *Enterococcus faecalis*. The mirtazapine + probiotic group demonstrated significantly greater changes in intestinal microbiota than mirtazapine alone, with larger increases in *Lactobacillus acidophilus* (*p* < 0.001) and *Bifidobacterium* (*p* < 0.001), and greater decreases in *Escherichia coli* (*p* < 0.001) and *Enterococcus faecalis* (*p* < 0.001) [33].

In terms of oxidative stress and neural plasticity, in pre-clinical studies, Juan et al. (2022) [27] reported that probiotics administration decreased hippocampal ROS levels significantly (*p* < 0.05) and significantly increased LTP, PSD95, and synaptophysin within the probiotic group (*p* < 0.0001). When comparing groups, probiotic group rats had significantly lower ROS levels (*p* < 0.0001), Iba-1 (*p* = 0.0004) and GFAP (*p* = 0.0012), and restored LTP (*p* < 0.001) compared to the placebo group [27]. Overall, these findings suggest that probiotics may provide therapeutic benefits by improving metabolic and nutritional status, modulating inflammation, modulating gut microbiota composition, and regulating metabolomic pathways.

### 3.4. Impact of Probiotics on Anxiety/Depression

Probiotics have shown promising effects on anxiety and depression in cancer. Sun et al. (2024) in a pre-clinical study reported that, compared with the CRC + CUMS group, kefir reduced depressive-like behavior, reflected by reduced immobility in the forced swim and tail suspension tests (*p* < 0.001 or *p* < 0.05), and improved anxiety-like behavior in the open-field and elevated plus maze tests (*p* < 0.001) [3]. In human studies, Tzikos et al. (2025) [17] demonstrated that within the probiotic group, depression and anxiety decreased significantly from baseline to the end of treatment and again at 3-month follow-up (depression: *p* < 0.001; anxiety: *p* = 0.009 at 1 month and *p* = 0.002 at 3 months). In contrast, the placebo group showed significant worsening of both depression (*p* = 0.002 to 1 month; *p* < 0.001 to 3 months) and anxiety (*p* < 0.001 to 3 months). When comparing groups, the probiotic group showed a markedly lower risk of remaining depressed at the end of treatment (RR = 0.18, 95% CI 0.10–0.31, *p* < 0.001) and at follow-up (RR = 0.10, 95% CI 0.05–0.19, *p* < 0.001). Similarly, the risk of persistent anxiety was significantly lower with probiotics at both time points (RR = 0.30, 95% CI 0.18–0.49, *p* < 0.001 at 1 month; RR = 0.13, 95% CI 0.07–0.24, *p* < 0.001 at follow-up) [17]. Similarly, Yang et al. (2016) [32] found that within the probiotics group, anxiety scores dropped significantly before surgery (*p* < 0.05), while scores in the placebo group increased. When comparing the probiotics group with the placebo group, the anxiety level was significantly lower in the probiotics group (*p* < 0.05) [32].

Other human studies that combined probiotics with other treatments reported beneficial effects of probiotics on anxiety and depression as well. Peng et al. (2021) [34] showed that both the probiotic and probiotic plus paroxetine groups had significant reductions in anxiety and depression scores (both *p* < 0.05). When the groups were compared, the combined group showed greater decreases in both measures (*p* < 0.001) [34]. Similarly, Lu et al. (2025) [33] reported that in the probiotic plus mirtazapine group, depression scores (SDS, HAMD, and BDI) decreased significantly from baseline (all *p* < 0.05). Compared with mirtazapine alone, the combined treatment produced significantly greater improvements across all depression scales at post-treatment (all *p* < 0.001) [33]. Also, Smoak et al. (2021) [35] reported a significant main effect of time for depression (BDI), indicating scores decreased from pre- to post-intervention across all participants (*p* = 0.032). A significant time × group interaction was also observed (*p* = 0.046), showing the reduction was greater in the kefir + exercise group (↓51.4%) compared with the exercise-only control (↓3.4%) [35]. Besides these promising results, Fitrikasari et al. (2024) [7] reported nonsignificant reductions in depression (*p* = 0.32) and anxiety (*p* = 0.91) within the probiotic group. Between groups, there were no significant differences in post-intervention depression (*p* = 0.508; 95% CI –1.6 to 3.1) or anxiety scores (*p* = 0.055) [7]. Similarly, Lee et al. (2014) [31] reported that PHQ-9 depression scores significantly improved within the probiotic group (*p* = 0.01), while no significant change occurred in the placebo group (*p* = 0.33); however, the between-group comparison did not reach statistical significance (*p* = 0.10) [31]. Consistent with these findings, Juan et al. (2022) in breast cancer patients reported no significant differences between the probiotic and placebo groups in anxiety or depression scores across the chemotherapy period [27].

### 3.5. Impact of Probiotics on Other Behavioral Outcomes

Across studies, probiotic interventions improved several behavioral outcomes beyond anxiety and depression, including stress, cognitive function, fatigue, sleep quality, and overall quality of life. Among studies that just evaluated the effects of probiotics alone, Fitrikasari et al. (2024) reported an overall decrease in stress-related DASS-42 scores within the probiotic group (*p* = 0.001), with a significant reduction in total DASS-42 scores compared to the placebo group (*p* = 0.048) [7]. Tzikos et al. (2025) [17] similarly reported that perceived stress (PSS-14) decreased significantly within the probiotic group from T1 (baseline) to T2 (end of treatment) and T3 (3-month follow-up) (*p* < 0.001), whereas stress increased significantly within the placebo group across the same time points (*p* < 0.001). Between groups, the probiotic group showed a significantly greater reduction in stress at T2 (RR = 0.51, *p* = 0.016) and an even more substantial reduction at T3 (RR = 0.06, *p* < 0.001). In the same study, sleep quality and insomnia symptoms improved significantly within the probiotic group from T1 → T2 → T3 (*p* < 0.001), while the placebo group exhibited deterioration or no improvement. Between-group comparisons confirmed that the probiotic group had significantly better sleep quality and fewer insomnia symptoms than the placebo group at both T2 (*p* < 0.001) and T3 (*p* < 0.05) [17]. Lee et al. (2014) [31] reported significant within-group improvements in colorectal cancer–related quality of life (FACT-C; *p* = 0.04) and fatigue (FACT-F; *p* = 0.02) among participants receiving probiotics. Between groups, the probiotic group demonstrated significantly greater improvements in functional well-being (FWB; *p* = 0.04) and FACT-C scores (*p* = 0.04) compared with placebo. Similarly, irritable bowel symptoms improved substantially in the probiotic group, decreasing from 67.9% at baseline to 45.7% after 12 weeks, whereas the placebo group showed little change (65.6% to 62.5%); this between-group difference was statistically significant (*p* = 0.03), with overall changes differing significantly between groups (*p* < 0.05) [31].

Juan et al. (2022) [27] reported significant between-group differences in HVLT-R IR (*p* = 0.003), HVLT-R DR (*p* = 0.001), BVMT-R IR (*p* = 0.001), BVMT-R DR (*p* = 0.003), visuospatial interference (*p* < 0.001), JoLO (*p* = 0.007), and VFT (*p* < 0.001). The total incidence of CRCI was also significantly lower in the probiotic group compared with placebo (35% vs. 81%; RR = 0.43, 95% CI 0.34–0.51; *p* < 0.001). Mild CRCI was reduced (29% vs. 52%; RR = 0.55, 95% CI 0.46–0.61; *p* = 0.003), and moderate CRCI was markedly lower (6% vs. 29%; RR = 0.22, 95% CI 0.05–0.30; *p* < 0.001) [27]. In the corresponding animal study by Juan et al. (2022), probiotics administration restored hippocampal LTP (*p* < 0.001), reduced oxidative stress (*p* < 0.0001), and enhanced synaptic plasticity markers (both *p* < 0.0001) compared to chemotherapy-only animals [27]. Sun et al. (2024) also reported that probiotic treatment in a colorectal cancer model reduced gastrointestinal inflammation and improved mucosal integrity (*p* < 0.001) [3] (Table 2).

**Figure 2:**
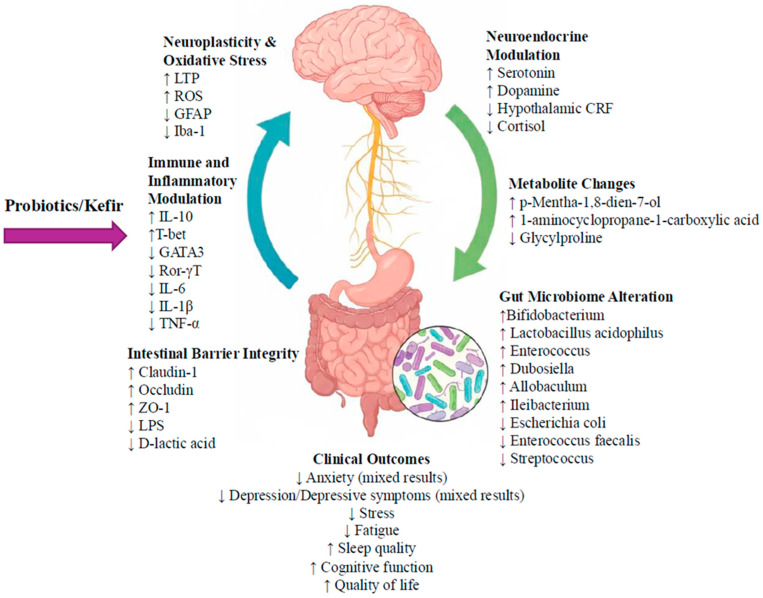
Schematic representation of the involved gut–brain axis after administration of probiotics/kefir in animal and human cancer studies (the image is representative and does not include all findings from included studies). ↓indicates a decrease; ↑indicates an increase. LTP: long-term potentiation, ROS: reactive oxygen species, GFAP: glial fibrillary acidic protein, Iba-1: ionized calcium-binding adaptor molecule 1, CRF: corticotropin-releasing factor, IL-10: interleukin-10, T-bet: T-box transcription factor TBX21, GATA3: GATA-binding protein 3, Ror-γT: retinoic-acid-related orphan receptor gamma t, IL-6: interleukin-6, IL-1β: interleukin-1 beta, TNF-α: tumor necrosis factor-alpha, LPS: lipopolysaccharide.

Among studies combining probiotics with other interventions, Lu et al. (2025) found that participants receiving probiotics plus mirtazapine experienced significant improvements in multiple quality-of-life domains, including dysphagia, stomach pain, dietary restriction, hiccups, anxiety, and overall symptom burden (all *p* < 0.05), with greater improvements than the mirtazapine only group (*p* < 0.001) [33]. Similarly, Smoak et al. (2021) [35] reported significant main effects of time for both fatigue (*p* = 0.017) and gastric (GI) distress (*p* = 0.013) across all participants. Significant time × group interactions were also observed for fatigue (*p* = 0.030) and GI distress (*p* = 0.021), indicating greater reductions in the kefir + exercise group compared with the exercise-only control [35] (Figure 2).

### 3.6. Safety and Tolerability

Across studies, probiotic administration was consistently reported as safe and well-tolerated with no serious adverse events [7,17,27,31,32,33,34,35]. Mild gastrointestinal symptoms were comparable between intervention and control groups. No participants withdrew due to intolerance of probiotics across trials.

## 4. Discussion

This systematic review revealed that probiotics were consistently safe and well-tolerated; however, their effects on anxiety and depression in cancer populations were inconsistent. In the animal studies, improvements in depressive- and anxiety-like behaviors occurred alongside measurable biological changes, suggesting mechanistic links. Reductions in hypothalamic CRF and corticosterone, restoration of hippocampal GR expression, improvements in microbial composition, and lowered pro-inflammatory cytokines (IL-6, IL-1β, TNF-α, IL-17) coincided with decreases in immobility and anxiety behaviors. These parallel biological and behavioral improvements highlight how modulation of the HPA axis, inflammation, oxidative stress, and microbiota composition may underlie the antidepressant- and anxiolytic-like effects of probiotics in pre-clinical models.

In contrast, results from human trials were more heterogeneous. Across studies, improvements in anxiety or depressive symptoms were observed in some probiotic groups but were not consistently superior to placebo. Importantly, trials that reported psychological improvement often also documented favorable biological changes, such as reductions in cortisol, improved inflammatory profiles, lower LPS or endotoxin markers, or increased serotonin or dopamine, suggesting a potential relationship between biomarker modulation and mental health outcomes. However, this pattern was not uniform across all studies, and several trials reported psychological improvement without clear biological change. Because interventions varied in probiotic composition, dosage, treatment duration, and cancer patient population, the available evidence does not allow conclusions about whether single-strain or multi-strain formulations are more effective to relief anxiety and depression.

### 4.1. Mechanisms of Probiotics Actions

Across animal and human studies, probiotics influenced several components of the gut–brain axis [3,7,27,33,34,35]. Probiotics improved microbial diversity and increased the relative abundance of beneficial taxa, such as *Lactobacillus*, *Bifidobacterium*, and *Enterococcus*, and reduced the relative abundance of potentially opportunistic/pathogenic taxa, such as *Escherichia coli*, *Enterococcus faecalis*, and *Streptococcus* [27,33]. Rebalancing of bacterial composition was accompanied by the production of metabolites, such as p-Mentha-1,8-dien-7-ol, linoelaidyl carnitine, and 1-aminocyclopropane-1-carboxylic acid, all of which are linked to anti-inflammatory effects [27]. Increased p-Mentha-1,8-dien-7-ol was observed in both breast cancer patients and rats, which was associated with reduced oxidative stress, improved hippocampal synaptic plasticity, and decreased microglial and astrocytic activation, supporting a neuroprotective role that probiotics can have within the gut–brain axis [27]. In studies where psychological improvement occurred, these microbial and metabolic changes generally moved in the same favorable direction, suggesting functional relevance, although a direct causal relationship cannot be established from the current evidence.

Probiotics also modulated systemic and neuroinflammatory pathways. In animal studies, kefir markedly reduced IL-6, IL-1β, TNF-α, and IL-17 in both serum and hippocampal tissue, restored T-cell transcriptional balance, and increased IL-10 [3,27]. Human studies produced more variable results; however, reductions in inflammatory or endotoxin markers, including lower LPS, D-lactic acid, and procalcitonin were reported in several trials [3,27,34,35]. Notably, in studies that documented psychological improvement, reductions in inflammatory burden often occurred in parallel. These cross-domain patterns support inflammation as a potential mediator but remain insufficient for definitive conclusions.

Neuroendocrine modulation was another pathway influenced by probiotics. In animal models, kefir reduced CRF and corticosterone and increased serotonin synthesis [3,27]. Human trials similarly reported reductions in cortisol or increases in serotonin or dopamine in some interventions, while others found no meaningful change [7,32,33]. Improvements in metabolic markers, such as glucose, LDL, ALB, Hb, and PA were also observed in certain studies and may reflect broader physiological stabilization [27,33,34]. Although these biological shifts did not consistently correspond with psychological outcomes across studies, several trials reported concurrent improvements, suggesting that neuroendocrine regulation may be one pathway through which probiotics influence anxiety and depression in cancer population [27,33]. Taken together, these findings point to several potential pathways through which probiotics may act, but they also highlight that future studies will need to use more rigorous designs and analyses to better separate the specific effects of probiotics from other factors that could influence outcomes.

### 4.2. Effects of Probiotics on Anxiety and Depression

Findings from animal studies showed that probiotic/kefir administration was associated with reductions in anxiety- and depressive-like behaviors, indicating potential therapeutic effects in controlled experimental settings [3,27]. In contrast, results from human studies were more variable. Some trials reported significant improvements in anxiety or depression, while others observed no differences from placebo [7,17,27,31,32,33,34,35]. These inconsistencies likely reflect substantial variation across studies in probiotic formulation, dosage, duration, cancer type, and treatment context. The available evidence does not allow us to draw firm conclusions about whether single-strain or multi-strain formulations are more effective for anxiety or depression in cancer populations. Notably, studies that combined probiotics with other interventions, such as antidepressants or exercise tended to show more pronounced improvements than probiotics alone, suggesting that adjunctive use may be more effective than standalone supplementation [33,34,35].

### 4.3. Effects of Probiotics on Other Behavioral Outcomes

Beyond anxiety and depression, probiotics have been studied for fatigue, sleep quality, gastrointestinal symptoms, cognition, and quality of life in cancer populations. Probiotics improved bowel symptoms and functional well-being in colorectal cancer survivors, reduced chemotherapy-related cognitive impairment in breast cancer patients, and enhanced fatigue and gastrointestinal distress outcomes when combined with exercise in survivors [17,33,35]. Probiotics also improved sleep quality in gastrointestinal cancer patients [17]. These results are consistent with prior evidence of probiotics reducing cancer-related gastrointestinal toxicities [36], cancer-related fatigue [37], and influencing sleep regulation via serotonin–tryptophan pathways [38].

### 4.4. Limitations

The present review has several limitations. We did not include gray literature sources such as ClinicalTrials.gov or preprint servers, which may contribute to publication bias and reduce the capture of unpublished or ongoing studies. Considerable heterogeneity existed across the included trials in probiotic strains, formulations, dosages, intervention durations, cancer types, treatment contexts, and outcome measures, which limits comparability and precluded conducting a quantitative meta-analysis. Many studies had modest sample sizes and, in some cases, insufficient reporting of randomization, allocation concealment, or blinding, increasing the risk of bias. In several studies, probiotics were administered alongside antidepressants or behavioral interventions, making it difficult to isolate their independent effects. Potential confounding factors such as diet, baseline microbiota composition, chemotherapy regimens, and disease stage were variably controlled or not addressed. Moreover, because included studies did not consistently exclude participants with comorbid physical or mental health conditions, the potential influence of comorbid conditions on outcomes could not be assessed, adding to the heterogeneity of study populations. Finally, most studies were short-term, which limits conclusions about both the durability of probiotic effects and their long-term safety.

### 4.5. Conclusions

This systematic review reveals that probiotics may reduce anxiety and depression in individuals with cancer, although the quality and consistency of evidence are variable. Single-strain probiotic formulations generally produced limited or inconsistent effects, whereas multi-strain probiotics were associated with greater reductions in anxiety and depression. The most noticeable effects occurred when probiotics were combined with pharmacological or behavioral interventions, such as antidepressant medications or exercise, suggesting a potential synergistic effect. Mechanistic evidence from animal and clinical studies shows that probiotics can restore gut microbiota composition and diversity, modulate systemic inflammation, stabilize neuroendocrine activity, including cortisol regulation, and influence tryptophan–serotonin metabolism. The findings indicate that the gut–brain axis is a key pathway linking probiotic to reductions in psychological distress. However, heterogeneity in probiotic strains, dosages, cancer types, and methodological quality across trials limits the ability to draw definitive conclusions. Larger, rigorously designed RCTs and mechanistic biomarker studies are needed to establish efficacy and determine optimal probiotic formulations for clinical application.

## Data Availability

No new data were created or analyzed in this study.

## Supplementary Materials

The following supporting information can be downloaded at: https://www.mdpi.com/article/10.3390/microorganisms14010051/s1, PRISMA 2020 checklist.
